# Supplementary material for: Inhibiting Methanogenesis in Rumen Batch Cultures Did Not Increase the Recovery of Metabolic Hydrogen in Microbial Amino Acids
Source: Microorganisms. 2019 Apr 27;7(5):115. doi: 10.3390/microorganisms7050115 (PMC6560395; doi:10.3390/microorganisms7050115)
Supplement: Supplementary file 1 [file microorganisms-07-00115-s001.pdf]

Table S1. Pairs of reducing equivalents ([2H]) produced or incorporated associated to the formation of volatile fatty acids and gases from glucose in rumen fermentation.

Metabolite	Overall stoichiometry	[2H] produced (mol/mol metabolite)	[2H] incorporated (mol/mol metabolite)
Acetate	$C_6H_{12}O_6 + 2\ H_2O \rightarrow 2\ CH_3COO^- + 2\ H^+ + 2\ CO_2 + 4\ [2H]$	2	0
Propionate	$C_6H_{12}O_6 + 2\ [2H] \rightarrow 2\ CH_3CH_2COO^- + 2\ H^+ + 2\ H_2O$	1	2
Butyrate	$C_6H_{12}O_6 \rightarrow CH_3CH_2CH_2COO^- + H^+ + 2\ CO_2 + 2\ [2H]$	4	2
Valerate	$C_6H_{12}O_6 + [2H] \rightarrow CH_3CH_2CH_2CH_2COO^- + H^+ + CO_2 + 2\ H_2O$	3	4
Methane	$CO_2 + 4\ [2H] \rightarrow CH_4 + 2\ H_2O$	0	4
Hydrogen	$[2H] \rightarrow H_2$	0	1

Table S2. Pairs of reducing equivalents ([2H]) produced and incorporated associated to the formation of amino acids from glucose, carbon dioxide (CO_2), preformed volatile fatty acids (VFA) and ammonium (NH_4^+) in rumen fermentation.

Equation	Amino acid	Overall stoichiometry of synthesis	[2H] produced (mol/mol amino acid)	[2H] incorporated (mol/mol amino acid)	Δ[2H] [(mol incorporated – mol produced)/mol amino acid]	Comment
1	Glu	$C_6H_{12}O_6 + 4\ CO_2 + 2\ NH_4^+ + 6$ [2H] → $2\ COO^-CH_2CH_2CHNH_3^+COO^- + 4\ H^+ + 6\ H_2O$	1	4	3	α-ketoglutarate produced by reverse tricarboxylic acid cycle (TCA)
2	Glu	$C_6H_{12}O_6 + NH_4^+ + CO_2$ → $COO^-CH_2CH_2CHNH_3^+COO^- + 2\ H^+ + 2\ CO_2 + 3$ [2H]	4	1	-3	α-ketoglutarate produced by forward TCA
3	Asp	$C_6H_{12}O_6 + 2\ NH_4^+ + 2\ CO_2$ → $2\ COO^-CH_2CHNH_3^+COO^- + 4\ H^+ + 2\ H_2O$	1	1	0	Oxaloacetate formed from pyruvate or phosphoenolpyruvate carboxylation
4	Ala	$C_6H_{12}O_6 + 2\ NH_4^+$ → $2\ CH_3CHNH_3^+COO^- + 2\ H^+ + 2\ H_20$	1	1	0	Pyruvate formed in glycolysis
5	Ala	$CH_3COO^- + CO_2 + NH_4^+ + 2$ [2H] → $CH_3CHNH_3^+COO^- + 2\ H_2O$	0	2	2	Pyruvate formed by reductive carboxylation of acetate added to the medium
6	Pro	$C_6H_{12}O_6 + 4\ CO_2 + 2\ NH_4^+ + 10$ [2H] → $2\ (CH_2)_3NHCHCOO^- + 4\ H^+ + 10\ H_2O$	1	6	5	Glu formed through Eq. 1
7	Pro	$C_6H_{12}O_6 + NH_4^+$ → $(CH_2)_3NHCHCOO^- + CO_2 + 2\ H_2O +$ [2H] $+ 2\ H^+$	4	3	-1	Glu formed through Eq. 2
8	Arg	$C_6H_{12}O_6 + 8\ NH_4^+ + 6\ CO_2 + 10$ [2H] → $2\ H_2N^+CNH_2NH(CH_2)_3CHNH_3^+COO^- + 6\ H^+ + 14\ H_2O$	2	7	5	Glu formed through Eq. 1

9	Arg	$C_6H_{12}O_6 + 4\ NH_4^+ \rightarrow$ $H_2N^+CNH_2NH(CH_2)_3CHNH_3^+COO^- + 3\ H^+ + 4\ H_2O + [2H]$	5	4	-1	Glu formed through Eq. 2
10	Ser	$C_6H_{12}O_6 + 2\ NH_4^+ \rightarrow$ $2\ HOCH_2CHNH_3^+COO^- + 2\ H^+ + 2\ [2H]$	2	1	-1	From 3-phosphoglycerate
11	Gly	$C_6H_{12}O_6 + 2\ NH_4^+ + 2\ H_2O \rightarrow$ $2\ CH_2NH_3^+COO^- + 2\ H^+ + 2\ CO_2 + 6\ [2H]$	4	1	-3	From ser. One [2H] pair released in formate from regeneration of methylene-THF to THF
12	Lys	$2\ C_6H_{12}O_6 + 4\ NH_4^+ + 4\ [2H] \rightarrow$ $2\ CH_2NH_3^+(CH_2)_3CHNH_3^+COO^- + 8\ H_2O + 2\ H^+$	2	4	2	From asp
13	Thr	$C_6H_{12}O_6 + 2\ NH_4^+ + 2\ CO_2 + 4\ [2H] \rightarrow$ $2\ CH_3CHOHCHNH_3^+COO^- + 2\ H^+ + 4\ H_2O$	1	3	2	From asp
14	Val	$C_6H_{12}O_6 + NH_4^+ \rightarrow$ $CH_3CHCH_3CHNH_3^+COO^- + H^+ + CO_2 + 2\ H_2O$	2	2	0	From glucose
15	Val	$CH_3CHCH_3COO- + CO_2 + NH_4^+ + 2\ [2H] \rightarrow$ $CH_3CHCH_3CHNH_3^+COO^- + 2\ H_2O$	0	2	2	Reductive carboxylation of isobutyrate followed by amination
16	Leu	$3\ C_6H_{12}O_6 + 2\ NH_4^+ \rightarrow$ $2\ (CH_3)_2CHCH_2CHNH_3^+COO^- + 6\ CO_2 + 2\ H_2O + 6\ [2H] + 2\ H^+$	5	2	-3	Glucose as sole carbon source
17	Leu	$C_6H_{12}O_6 + CH_3COO^- + NH_4^+ \rightarrow$ $(CH_3)_2CHCH_2CHNH_3^+COO^- + 2\ CO_2 + 2\ H_2O + [2H]$	3	2	-1	Carbons 1 and 2 contributed by preformed acetate
18	Leu	$CH_3CHCH_3CH_2COO^- + CO_2 + NH_4^+ + 2\ [2H] \rightarrow$ $(CH_3)_2CHCH_2CHNH_3^+COO^- + 2\ H_2O$	0	2	2	Reductive carboxylation of isovalerate followed by amination
19	Ile	$C_6H_{12}O_6 + NH_4^+ + 3\ [2H] \rightarrow$ $CH_3CH_2CHCH_3CHNH_3^+COO^- + 4\ H_2O + H^+$	2	5	3	Glucose as sole carbon source
20	Ile	$CH_3CH_2CHCH_3COO- + CO_2 + NH_4^+ + 2\ [2H] \rightarrow$ $CH_3CH_2CHCH_3CHNH_3^+COO^- + 2\ H_2O$	0	2	2	Reductive carboxylation of 2-methylbutyrate followed by amination

21	Tyr	2 $C_6H_{12}O_6$ + NH_4^+ → $C_9H_{11}NO_3$ + 3 CO_2 + 3 H_2O + H^+ + 5 [2H]	7	2	-5	Shikimate cycle. Erythrose-4-phosphate from pentose cycle
22	Phe	2 $C_6H_{12}O_6$ + NH_4^+ → $C_9H_{11}NO_2$ + 3 CO_2 + 4 H_2O + H^+ + 4 [2H]	6	2	-4	Shikimate cycle. Erythrose-4-phosphate from pentose cycle
23	His	$C_6H_{12}O_6$ + H_2O + ATP + 2 NH_4^+ → $C_6H_9N_3O_2$ + 5-aminoimidazole-4-caboxamide ribonucleotide + CO_2 + 3 [2H]	3	0	-3	Phosphoribosyl pyrophosphate from pentose cycle.
